# Isolation of Reovirus T3D Mutants Capable of Infecting Human Tumor Cells Independent of Junction Adhesion Molecule-A

**DOI:** 10.1371/journal.pone.0048064

**Published:** 2012-10-24

**Authors:** Diana J. M. van den Wollenberg, Iris J. C. Dautzenberg, Sanne K. van den Hengel, Steve J. Cramer, Raoul J. de Groot, Rob C. Hoeben

**Affiliations:** 1 Department of Molecular Cell Biology, Leiden University Medical Center, Leiden, The Netherlands; 2 Virology Division, Department of Infectious Diseases and Immunology, Faculty of Veterinary Medicine, Utrecht University, Utrecht, The Netherlands; McMaster University, Canada

## Abstract

Mammalian Reovirus is a double-stranded RNA virus with a distinctive preference to replicate in and lyse transformed cells. On that account, Reovirus type 3 Dearing (T3D) is clinically evaluated as oncolytic agent. The therapeutic efficacy of this approach depends in part on the accessibility of the reovirus receptor Junction Adhesion Molecule-A (JAM-A) on the target cells. Here, we describe the isolation and characterization of reovirus T3D mutants that can infect human tumor cells independent of JAM-A. The JAM-A-independent *(jin*) mutants were isolated on human U118MG glioblastoma cells, which do not express JAM-A. All *jin* mutants harbour mutations in the S1 segments close to the region that encodes the sialic acid-binding pocket in the shaft of the spike protein. In addition, two of the *jin* mutants encode spike proteins with a Q336R substitution in their head domain. The *jin* mutants can productively infect a wide range of cell lines that resist *wt* reovirus T3D infection, including chicken LMH cells, hamster CHO cells, murine endothelioma cells, human U2OS and STA-ET2.1 cells, but not primary human fibroblasts. The *jin*-mutants rely on the presence of sialic-acid residues on the cell surface for productive infection, as is evident from wheat germ agglutinin (WGA) inhibition experiments, and from the *jin-*reovirus resistance of CHO-Lec2 cells, which have a deficiency of sialic-acids on their glycoproteins. The *jin* mutants may be useful as oncolytic agents for use in tumors in which JAM-A is absent or inaccessible.

## Introduction

The *Reoviridae* constitute a family of viruses with a non-enveloped icosahedral capsid and a segmented double-stranded RNA genome. Prototypes of the mammalian Orthoreoviruses were isolated from the human respiratory and enteric tracts and have not been associated with serious human disease. The human reovirus type 3 Dearing (T3D) is frequently studied and often serves as a model for the family. The reoviruses have a lytic replication cycle and preferentially induce cell death and apoptosis in tumor cells but not in diploid, non-transformed cells [1]–[3]. In transformed cells reovirus uncoating and replication are stimulated [4]–[8]. In addition, Ras signalling sensitizes the cells to reovirus-induced apoptosis [9]. Based on these observations, reovirus T3D is a promising candidate for use as oncolytic agent, and is currently evaluated in a variety of clinical cancer therapy trials [10]–[13].

Reovirus attachment to cells is a multi-step process. The reovirus spike protein σ1 binds with a region of its shaft domain to cell surface-bound sialic acids with low-affinity, before the head domain of σ1 engages the high affinity receptor Junction Adhesion Molecule-A (JAM-A, also known as JAM-1) [14], [15]. Following receptor binding, virions become internalized by a mechanism involving the capsid protein λ2 binding to β1 integrins [16], [17]. An alternative entry pathway can be employed upon proteolytic removal of the reovirus outer capsid protein σ3 and cleavage of μ1/µ1C, yielding intermediate (or infectious) subviral particles (ISVPs). The ISVPs can directly penetrate the cellular membrane independent of the presence of JAM-A [18], [19]. The ISVPs are similar to the disassembly intermediates formed during cellular entry via the endocytosis pathway.

The reovirus receptor JAM-A is expressed in epithelial and endothelial cells of several tissues including lung, kidney, pancreas, heart, brain, intestine and lymph nodes [20] but some tumor cells have down-regulated the JAM-A receptors on their cell surface, thereby limiting the susceptibility to reovirus T3D. JAM-A expression was found significantly down-regulated in clear-cell renal carcinoma cells [21]. Also, cells grown from freshly isolated colorectal tumor metastases resist reovirus infection. Immunohistochemistry demonstrated that JAM-A is not accessible at the cell surface, although JAM-A is detectable intra-cellularly [22]. Furthermore, there is an inverse correlation of JAM-A expression in breast cancer cells and their ability to migrate. JAM-A is expressed in normal human mammary epithelial cells but in metastatic breast cancer tumors the expression is down-regulated [23].

Here we describe the isolation and characterization of reovirus T3D mutants that are adapted to propagation in JAM-A negative, reovirus-T3D resistant cell lines. The first was identified as a spontaneously occurring mutant in one of our batches genetically retargeted reovirus [24]. Subsequently two other mutants were isolated by selection on JAM-A negative human glioblastoma cells. We demonstrate that these JAM-A-independent *(jin*) mutants employ an as yet unidentified, but apparently ubiquitous receptor, which is present on a wide variety of cell types. Their potential use as novel oncolytic tools against tumor cells in which JAM-A is absent or inaccessible is discussed.

## Results

### Isolation of a JAM-A Independent Reovirus Mutant

Previously we described a system for generating genetically modified reoviruses. The modification strategy relies on the exchange of a genome segment encoding the spike protein σ1 by a segment encoding a his-tagged spike. The modified viruses can be selected and propagated on U118scFvHis cells. This cell line is a derivative of the JAM-A negative human glioblastoma cell line U118MG and expresses a single-chain Fv (scFv) on its surface that is capable of binding the His-tag. The scFv serves as an artificial receptor for the σ1-His containing viruses [24].

In one of the batches of σ1-His modified reoviruses, we noted that a cytopathic effect (CPE) was not only induced in the U118scFvHis cells, but also in the parental U118MG cells. This suggested that this batch contains viruses that are capable of infecting cells independent of the presence of JAM-A and independent of the artificial scFv-His receptor.

**Figure 1:**
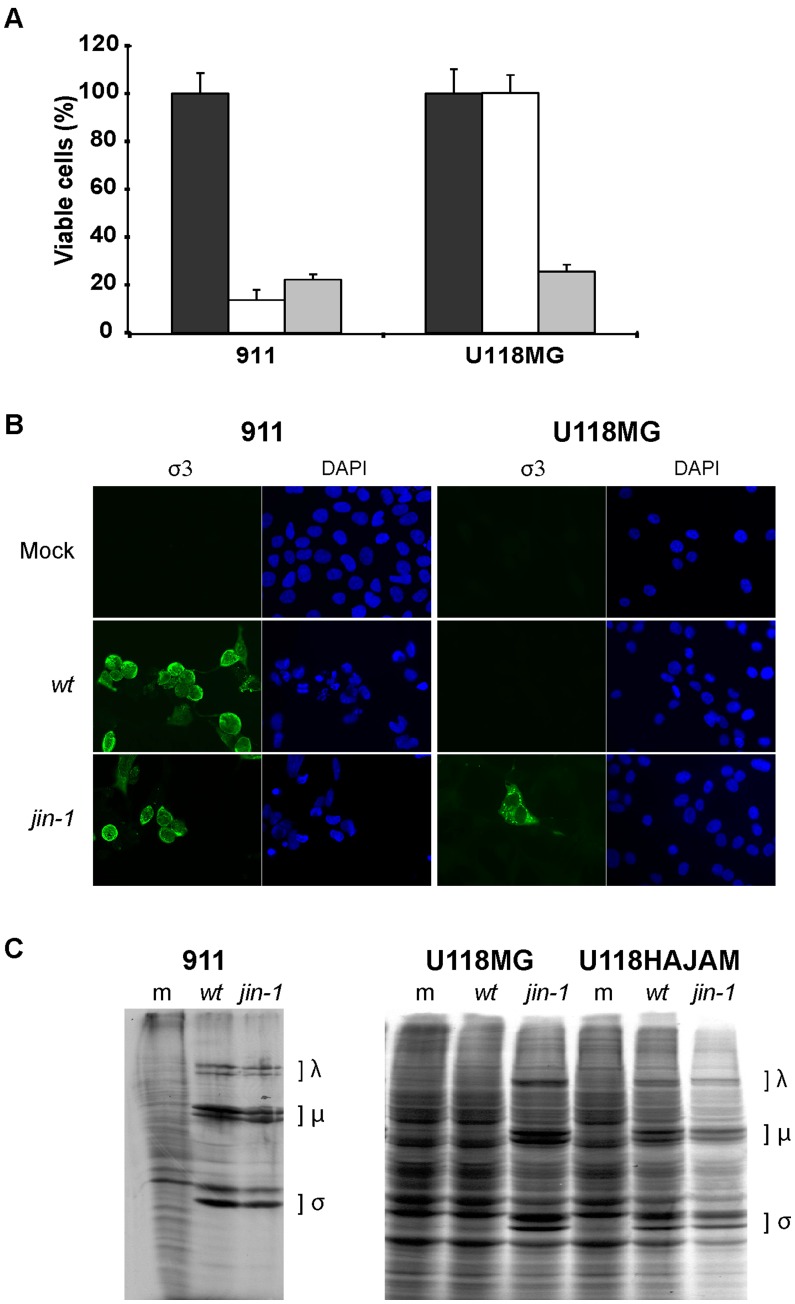
Reovirus mutant *jin-1* is able to infect the JAM-A negative cell line U118MG. (A) Viability assay (WST-1) on 911 and U118MG cells. Cells were mock infected (black bar) or infected with *wt* T3D (white bar) or *jin-1* virus (grey bar) with an MOI of 10, six days post infection. Means (± standard deviation) from three wells. (B) Detection of outer capsid protein σ3 in 911 and U118MG cells after addition of *wt* T3D or *jin-1* virus with an MOI of 5. 40 hr post infection cells were stained with a monoclonal antibody directed against σ3 (4F2) and visualised with a fluorescein isothiocyanate (FITC)-conjugated goat-anti-mouse secondary antibody. The nuclei are visualised with 4′,6-diamidino-2-phenylindole (DAPI). (C) Assessment of reoviral protein synthesis in *jin-1* or *wt* T3D infected cells. Indicated cells were infected with *wt* T3D or *jin-1* virus and labeled with [^35^S]-methionine once CPE became apparent. 911 cells were infected with an MOI of 1 and U118MG or U118HAJAM cells with MOI of 5. Indicated are the positions of the reoviral λ, µ and σ proteins. m represents mock infected cells; *wt*: *wt* T3D infected cells and *jin-1*: *jin-1* infected cells.

The first mutant virus isolated, which was called *jin-1* (JAM-A independent), was further propagated on U118MG cells. The *jin-1* mutant virus was compared to our lab reference *wt* T3D reovirus. In contrast to the *wt* T3D reovirus, the *jin-1* virus induces CPE in U118MG cells as is evident from a WST-1 cell viability assay (Fig. 1A). Both viruses are equally cytolytic to 911 cells which do contain JAM-A [24]. Immunofluorescence assays using an antibody against the major capsid protein σ3 confirmed the presence of σ3 in U118MG cells infected with *jin-1*, but not with *wt* T3D (Fig. 1B). To further verify that U118MG cells support the replication of the *jin-1* mutant, a metabolic labelling with [^35^S]-methionine was performed. As a positive control the U118-HAJAM cell line was included. This U118MG-derived cell line had been transduced with a lentivirus to overexpress an HA-tagged version of the JAM-A receptor. In U118-HAJAM and in 911 cells, exposure to the *wt* T3D as well as to *jin-1* reoviruses established infection as is evident from the synthesis of reovirus proteins. In contrast, in U118MG cells the reoviral protein synthesis was only detectable upon infection with the *jin-1* virus but not with our *wt* T3D (Fig. 1C). These data demonstrate that the *jin-1* reovirus, in contrast to *wt* T3D, is capable of infecting and replicating in the JAM-A negative cell line U118MG.

### Sequence Analysis of the Reovirus Mutants

The *jin-1* mutant originated from the U118scFvHis cell line. Since this mutant can infect JAM-A negative U118MG cells, we speculated that the attachment protein σ1 was altered. After one round of plaque purification and further propagation for eleven passages on U118MG cells, the complete genome was sequenced. The primers used (for this) are listed in Table S1. The PCR products were purified and used for sequence analysis. In the S1 segment two mutations occurred. The mutation led to a threonine-to-methionine change at position 193 (T193M) and a glutamine-to-arginine change at position 336 of the protein (Q336R). Also in other segments mutations were found (table 1).

**Table 1:** Amino acid differences in reovirus proteins of the *jin* mutants and the *wt* T3D reovirus strain.

RNA segment (protein)	AA position	*wt*	*jin-1*	*jin-2*	*jin-3*
**S1 (σ1)**	**187**	Gly		Arg	
	**193**	Thr	Met		
	**196**	Gly			Arg
	**336**	Gln	Arg	Arg	
**S2 (σ2)**	**254**	Ser	Phe		
**S3 (σNS)**	**No changes**				
**S4 (σ3)**	**177**	Ser	Phe		
	**198**	Gly	Glu		
	**357**	Met	Thr		
**M1 (μ2)**	**No changes**				
**M2 (μ1)**	**388**	Lys	Arg		
	**530**	Thr	Ala		
**M3 (µNS)**	**705**	Ala	Val		
	**706**	Asp	Ala		
	**708**	Val	Ala		
**L1 (λ3)**	**413**	Ile	Ser		
**L2 (λ2)**	**1101**	Met	Ile		
**L3 (λ1)**	**201**	Thr	Ala		
	**703**	Arg			Gly
	**1164**	Ser			Phe

To expand the pool of mutants, we repeated the procedure and exposed the U118scFvHis cells with our wild-type virus before further expansion on U118MG cells. After the first selection rounds in the U118scFvHis cells, we again found the Q336R mutation in the σ1 head domain. Upon prolonged propagation (10 passages) on U118MG cells an additional mutation was found in S1, resulting in a G187R change. This mutant strain, carrying mutations resulting in a Q336R and G187R change was named *jin-2.* Based on the findings in *jin-2* S1 in the earlier passage, we analyzed S1 of an earlier passage of *jin-1* as well (prior to plaque purification) and also in this S1 segment the only mutation present was the one resulting in the Q336R change in σ1.

**Figure 2:**
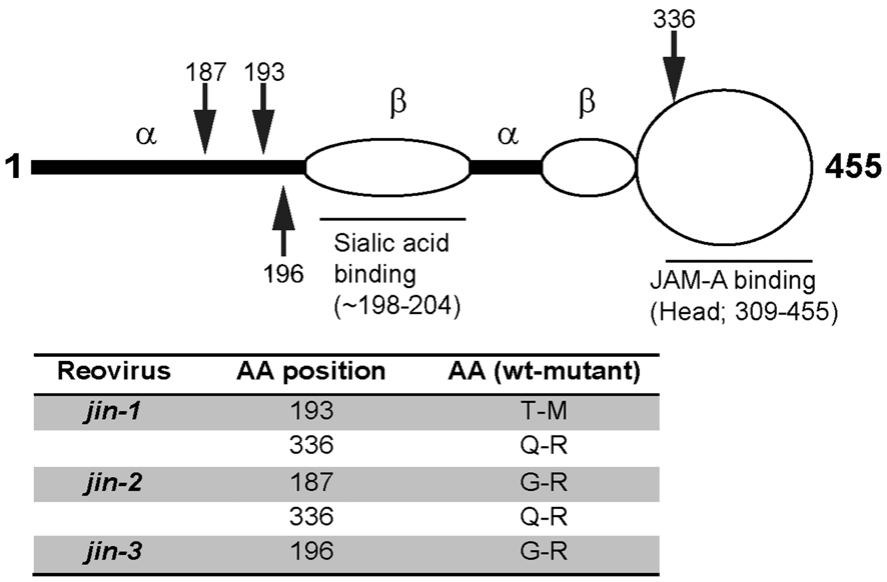
Schematic representation of mutations in Sigma-1. Model of the σ1 protein (adapted from Chappel et al, 2009). Arrows indicate the positions of the mutations.

Another mutant reovirus (*jin-3*) was obtained after direct exposure of U118MG cells at very high MOI to *wt* T3D reovirus. This virus was blindly passaged (i.e. the cells were lysed without signs of overt CPE at the time of virus harvest) for 6 rounds on U118MG cells. After 6 rounds in U118MG cells, CPE became apparent. After plaque purification on 911 cells and 10 additional passages on U118MG the complete genome of the *jin*-3 mutant was sequenced. Only one mutation was found in the S1 segment, resulting in a G196R alteration. Table 1 gives a summary of all the amino acid changes found in the mutants, compared to our *wt* T3D. A schematic overview of the amino acid changes in σ1 is depicted in Figure 2. In all *jin* mutants the amino-acid alterations in the shaft of σ1 are located close to the sialic acid (SA) binding motif [6], [15], [25], [26].

### Primary Human Fibroblasts (VH10 Cells) do not Support Replication of the *jin* Mutants or wtT3D

**Figure 3:**
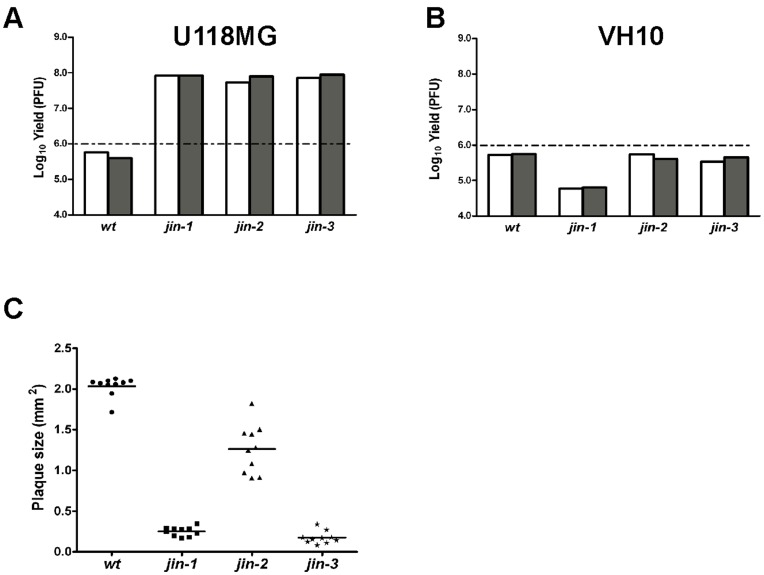
Comparison of wt T3D with *jin*-viruses in terms of plaque size, yield in U118MG cells and primary human fibroblasts (VH10 cells). (A) Yield of *wt* T3D and *jin-1*, *jin-2* and *jin-3* mutants from U118MG cells infected with MOI 10 per virus. Yields were determined 72 hours post infection by plaque assays on 911 cells. The graph shows yields (Log_10_ PFU) of two independent U118MG cell infections: first one is shown as a white bar and second as a grey bar. The dashed line represents the input amount of the initial infection. (B) Yield of *wt* T3D and *jin-1*, *jin-2* and *jin-3* mutants from VH10 cells infected with MOI 10 per virus. Yields were determined 72 hours post infection by plaque assays on 911 cells. The graph shows yields (Log_10_ PFU) of two independent VH10 cell infections: first one is shown as a white bar and second as a grey bar. The dashed line represents the input amount of the initial infection. (C) Plaque sizes of *wt* T3D and *jin-1*, *jin-2* and *jin-3* mutants in 911 cells. Surface areas of 10 plaques per virus were measured four days post infection with Olympus DP-software.

To study whether the *jin* mutants acquired the capacity to replicate in normal, non-transformed human cells, we exposed diploid human foreskin fibroblasts (VH10 cells) to *wt* T3D and to the *jin* mutants. The skin fibroblasts were chosen because primary human fibroblasts do not express JAM-A [27]. We studied the yields of the *jin* viruses and compared these with the yields of *wt* T3D on VH10 fibroblasts and on U118MG cells. As expected U118MG cells yielded high titers of the *jin* reoviruses, while *wt* T3D virus yields were below the amounts of virus added to the cells (Fig. 3A). On VH10 fibroblasts, neither the *wt* T3D reovirus nor the three *jin*-mutants yielded significant titers (Fig. 3B). Furthermore in the VH10 cell cultures no apparent signs of cell death were observed (data not shown). These data suggest that, like *wt* T3D our *jin* mutants do not replicate in normal non-transformed diploid fibroblasts.

During the plaque assays on 911 cells for the determination of viral yields, we noted that the plaques formed by the *jin-1* virus and *jin*-3 virus were consistently smaller than those of the *wt* T3D virus; the plaque surface area of the initial *jin-1* virus and *jin-3* virus is approximately 10-fold lower (Fig. 3C), suggesting reduced cell-to-cell spread of the mutants.

The *jin-2* virus also has a reduced plaque size compared with the *wt* virus, but the variation within the population is larger, which suggests heterogeneity in the population. Sequence analysis of the S1 segment of both the smaller and the larger *jin-2* plaques revealed that the smaller plaques contained the mutations for the G187R and Q336R change, while the larger plaques only contained the mutation that yield the Q336R alteration in σ1 (data not shown).

### Protease Inhibitor E64d Blocks Entry of *jin*-1 Virus

**Figure 4:**
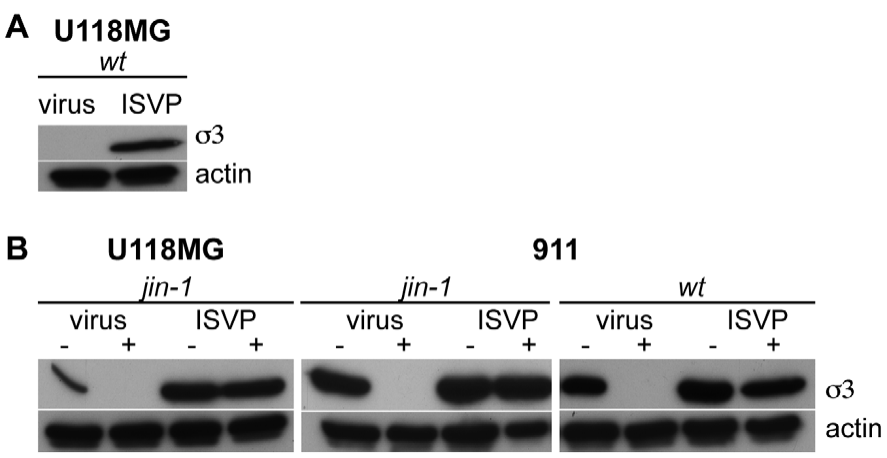
Effect of the cysteine protease inhibitor E64d on *jin*-1, *wt* T3D virus, and ISVP entry into cells. (A) U118MG cells were exposed to purified *wt* T3D virus and *wt* T3D ISVP (2*10^3^ particles per cell). Lysates were made 24 hours post-infection and analyzed by SDS-PAGE and western-blotting. The reovirus σ3 proteins were detected by the anti-reovirus σ3 antibody 4F2 and an anti-Actin serum was used to detect actin as a loading control. (B) Effect of 100 µM E64d on the entry of particles compared to entry of ISVPs. Cells, treated (+) or untreated (−) with 100 µM E64d, were exposed to *jin-1* (U118MG and 911 cells) or *wt* T3D (911 cells) virus or ISVPs (2*10^3^ particles per cell). Lysates were made 24 hours post-infection. Equal amounts of protein were loaded on 10% SDS-polyacrylamide gel and detected with anti-reovirus σ3 antibody (4F2), and anti-Actin as a loading control.

Reoviruses enter cells by receptor-mediated endocytosis after attachment of the σ1 protein to the JAM-A receptor [16], [28]. Subsequently, the viral λ2 protein binds cellular integrins leading to endocytosis. In the endosomes the particle undergoes conformational changes by partial proteolysis, leading to intermediate subviral particles (ISVPs). The outer capsid proteins σ3 and µ1/µ1C are cleaved by cellular proteases and σ1 undergoes a conformational change. *In vitro*, this process can be mimicked by proteolytic treatment of complete virions. The generated ISVPs are capable to enter cells independent of the JAM-A receptor by penetration of the cytoplasmic membrane [18], [19], [29]. One possible explanation for the JAM-A independent entry of the *jin-1* virus could be a premature transition to ISVPs prior to entry into the cell. To test whether the *jin-1* virus is still dependent on cellular proteases the protease inhibitor E64d was used. If cells are exposed to E64d prior to infection, intact virions are trapped in the endosome while ISVPs can complete the replication cycle [30], [31]. To confirm that *wt* ISVPs are JAM-A independent we exposed the U118MG cells to *wt* T3D ISVPs and to intact T3D virions (fig. 4A). As expected, only in the cells exposed to the ISVPs, viral protein σ3 synthesis is detected, evidencing virus entry and viral protein synthesis. In 911 cells both the *jin*-*1* virus and *wt* T3D virus are inhibited by E64d (Fig. 4B), while σ3 was detected in both the *jin-1* and *wt* ISVP infected cells. Also in the U118MG cells, the *jin-1* virus entry is blocked by the presence of E64d, but not the *jin-1* ISVP entry. These data demonstrate that like *wt* T3D reovirus, the reovirus mutant *jin*-*1* exploits the endocytotic pathway to enter U118MG cells.

### *jin-1* σ1 Forms Trimers

**Figure 5:**
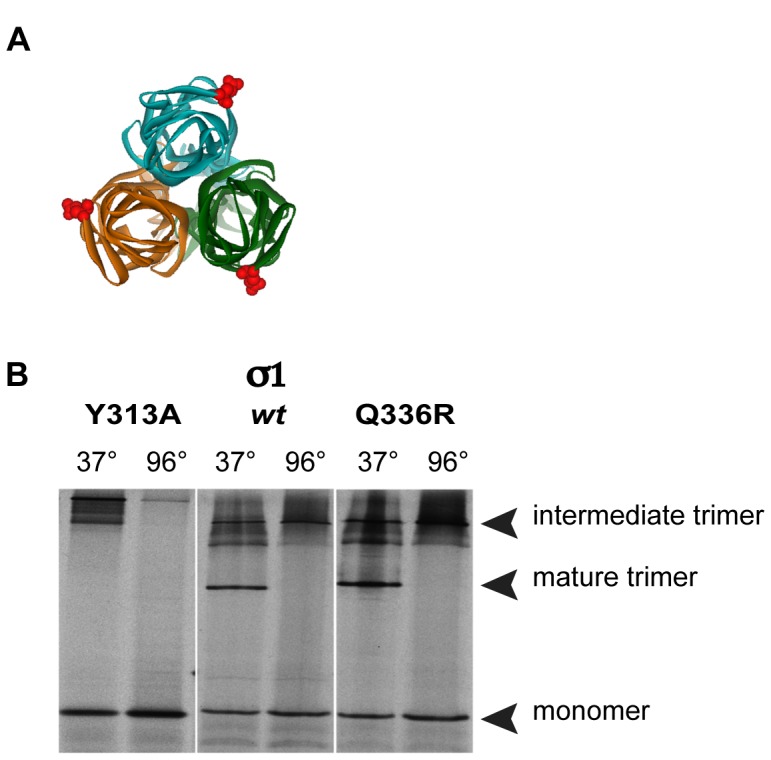
Analysis of Sigma-1 trimers synthesized *in vitro.* (A) Top view of σ1-trimer, with colored monomer units (green, turquoise, orange). Position of the Q336R mutation in each monomer is indicated as red CPK symbol (Chappell et al., 2002); PDB ID: 1KKE. The software used for the 3D graphs is Viewerlite 5.0 from Accelrys. (B) [^35^S] methionine labelled *in vitro* transcribed and translated products of plasmids pDGC-S1wt (S1wt), pDGC-S1Q336R (S1Q336R) and pDGC-S1Y313A (S1Y313A) were incubated for 30 minutes at 37°C (to stabilize the mature trimers) or boiled for 5 minutes (to disrupt the trimers), before loading on a 10% SDS-polyacrylamide gel at 4°C. The position of the three different conformations is indicated.

The change at amino-acid position 336 is located close to the domain that has been implicated in trimerization of σ1 [32]. Although the Q336R alteration is located at the outward surface-exposed side of every monomer in the trimeric conformation (Fig. 5A, R336 is shown in red) it is essential to confirm that the Q336R alteration does not interfere with trimer formation. To this end an *in vitro* trimerization assay was performed as described by Leone at al. [33]. For this *wt* σ1, σ1-Q336R and σ1-Y313A proteins were synthesized *in vitro*. The Y313A change abolishes the capacity of σ1 to form trimers [32]. The σ1 products were analysed by mild PAGE at 4°C (Fig. 5B). Whereas the σ1-Y313A protein does not form mature trimers, both the *wt* T3D σ1 and σ1-Q336R do. Intermediate trimers, which consist of σ1 molecules in which only the shaft is trimerized while the head domain is in a monomeric configuration, are detectable in all σ1 variants tested. Our data show that the Q336R alteration that occurs in the *jin-1* and *jin-2* viruses does not affect the formation of mature σ1-trimers *in vitro*.

### *jin-1* and *jin-2* have Selective Advantages Over Wild-type Reovirus in U118MG Cells

**Figure 6:**
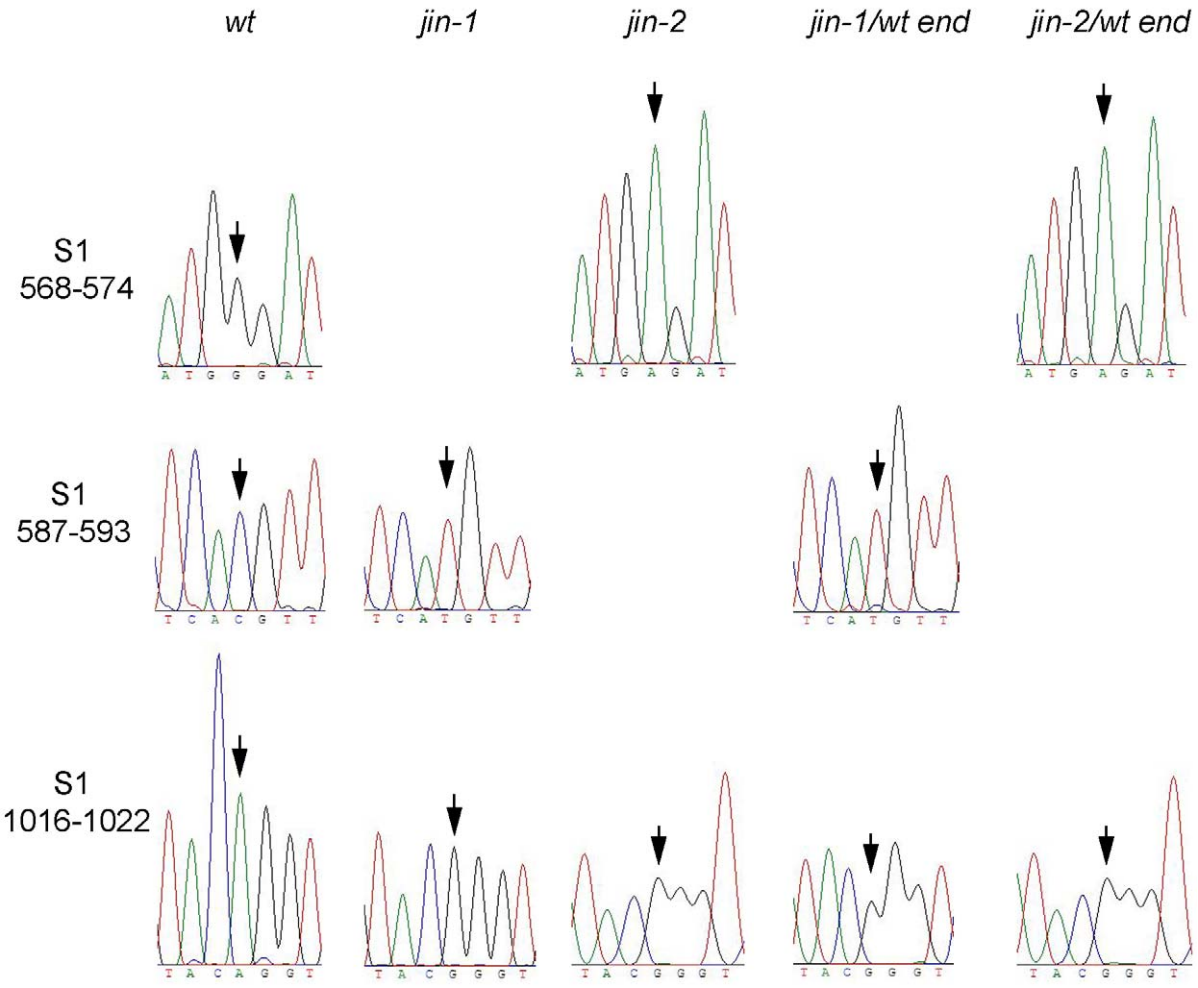
S1-sequence analysis of the *jin-1*/*wt* T3D and *jin-2*/*wt* T3D selection assay on U118 MG cells. The *jin-1* or *jin-2* viruses were mixed with a 100-fold excess of *wt* T3D virus with regards to MOI. 911 cells were exposed to the mixtures first, before propagation on U118MG cells for three more passages. Reovirus RNA was isolated from the virus derived from the third passage on U118MG cells and subjected to RT-PCR to obtain the S1 products from the total population (*jin-1/wt* end or *jin-2/wt* end). Sequence histograms of the indicated regions were compared to the S1 sequences of the input reoviruses. Arrows indicate the nucleotide differences between the *wt* T3D and *jin-1* or jin*-2*. (S1 nucleotide positions 571, 590 and 1019).

To confirm that *jin-1* and *jin-2* viruses have a selective advantage over *wt* T3D in U118MG cells, we mixed *jin-1* or *jin-2* with a 100-fold excess of *wt* T3D prior to infection of cells. Cultures of 911 cells were infected at an MOI 10 with the mixtures to allow reassortment of genome segments to take place. Two days post-infection, the virus was harvested by three freeze-thaw cycles and used to infect U118MG cells. While no CPE for *jin-1/wt* mixtures was observed at 7 days post-infection in the U118MG cells, the cells were freeze-thawed and the lysate was used to infect fresh U118MG cultures. In U118MG cells infected with *jin-2/wt* T3D mixture the virus was harvested after 4 days, with visible signs of CPE. This procedure was repeated for two more times. At passage 3 clear CPE was observed four days post infection in both *jin-1/wt* and *jin-2/wt* selections. Sequencing of PCR products after reverse transcription PCR of the S1 segment after the third selection on U118MG cells were compared with the S1 sequences of *wt*T3D, *jin-1* and *jin-2* (Fig. 6). S1 of the *jin-1/wt* end population contains a T at nucleotide position 590 and a G at position 1019, identical to the *jin-1* S1 segment. Sequence results for S1 of the *jin-2/wt* end population revealed an A at position 571 and a G at position 1019 and this is identical to *jin-2* S1 sequence. From these findings we conclude that in U118MG cells σ1 proteins from *jin-1* or *jin-2* provide a strong selective advantage over *wt*T3D σ1. These data provide evidence that the amino-acid alterations in σ1 provide the *jin* mutants with the capacity to infect and replicate in JAM-A-negative cells.

### *jin-1* Reoviruses Infect Cells that are Non-permissive for *wt* T3D

**Figure 7:**
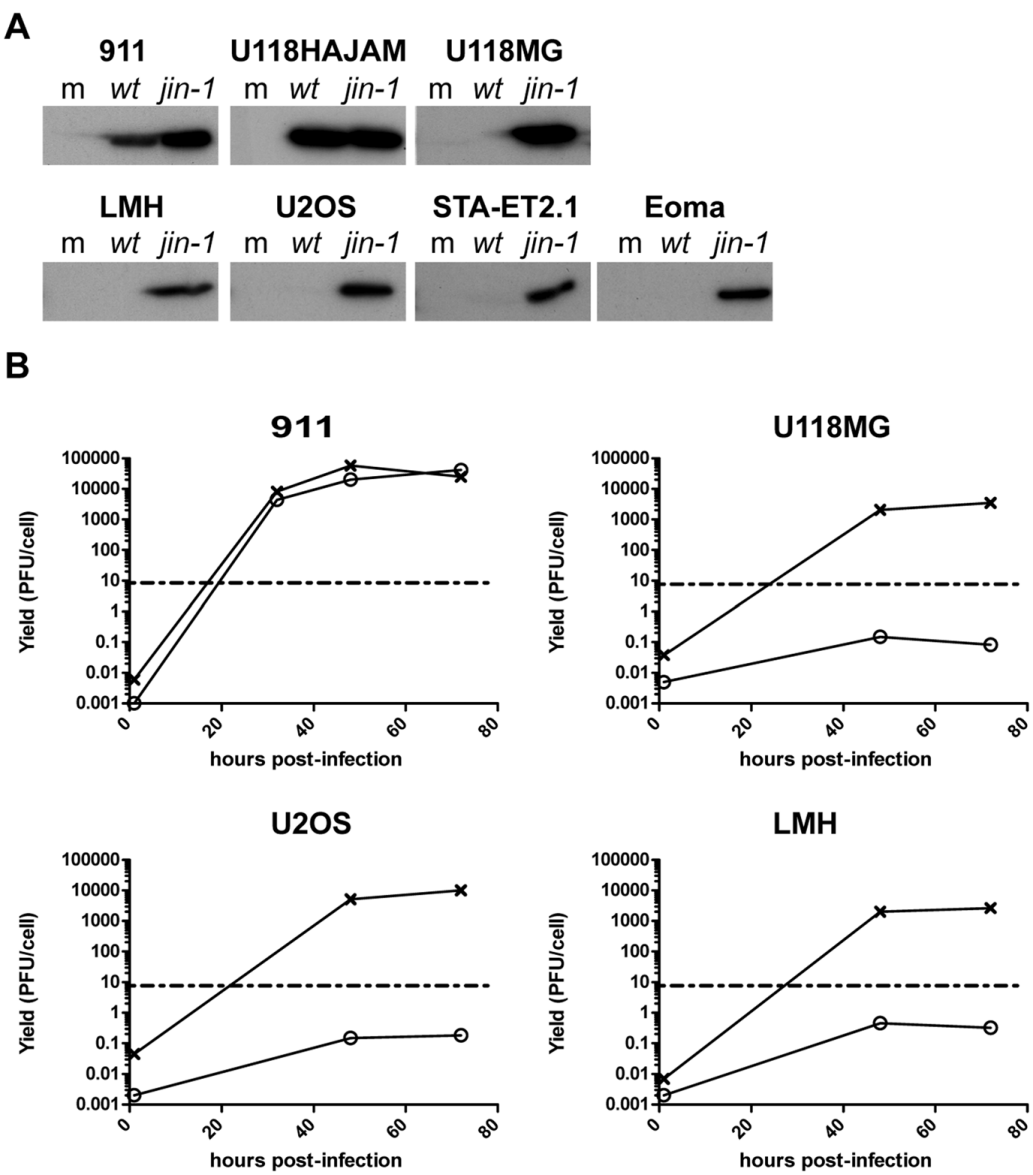
Reovirus mutant *jin-1* can infect cell lines that resist *wt* T3D reovirus infection. (A) Several cell lines were infected with *wt* T3D or *jin-1* and 32 hr post-infection cells were lysed. Protein samples (30 µg) were analyzed by 10% SDS-polyacrylamide gel electrophoresis. For the immunodetection anti-reovirus σ3 (4F2) was used. The cell lines 911 and U118-HAJAM are included to serve as positive controls for the infectivity of *wt* T3D. The cells were mock-infected (m); *wt* T3D infected, or *jin-1* infected. (B) Virus production of *wt* T3D and *jin-1* in the different cell lines. Cells were exposed to virus at MOI of 10 for one hour, washed with PBS and immediately lysed (1 hour time point) or left for 48 or 72 hours. For 911 cells an additional harvest point at 32 hours post-infection was included. The viral titers in the samples were determined by plaque assays on 911 cells. The graph shows a representative example of the assay. Open circles: *wt* T3D(o), crosses: *jin-1*(x). The dashed line represents the input amount of the initial infection (10 PFU/cell).

To study whether the *jin-1* mutant has expanded its tropism beyond the U118MG cell line, we evaluated whether this virus can replicate in a panel of cell lines that resists infection with *wt* T3D virus. These cell lines include chicken hepatoma cell line LMH [34], murine endothelioma cell line Eoma [35], human bone osteosarcoma cell line U2OS [36] and human Ewing sarcoma cell line STA-ET2.1 [37]. In parallel the cell lines 911 and U118-HAJAM were included as positive controls for infection. Each of the cell lines were exposed to *wt* T3D or *jin-1* viruses with an amount of virus corresponding to 8 PFU/cell as determined in 911 cells. While no major capsid protein σ3 was detected in the *wt* T3D-resistant cell lines exposed to wt T3D, exposure of these cells to *jin-1* resulted in the detection of the σ3 protein at 36 hr post infection (Fig. 7A). In 911 and U118HA-JAM cells, the σ3 protein is present in *wt* T3D infected cells as well as in the *jin-1* infected cells.

To verify that *wt* T3D resistant cells could support replication of the *jin*-1 virus, the virus yields were determined in some of these cell lines (Fig. 7B). In 911 cells both viruses give a similar yield, but in three other cell lines (U118MG, U2OS and LMH) more progeny virus was produced with the *jin-1* virus than with *wt* virus. The amount of *wt* T3D produced per cell did not rise above the amount added to the cells (MOI of 10, dashed lines). From these data we conclude that the *jin-1* virus is able to productively infect our panel of *wt* T3D resistant cells.

### Cell Entry of Reovirus *jin-1* and *jin-3* Relies on Sialic Acids

Apart from the Q336R mutation, the other mutations found in the S1 segments of the *jin* mutants are located close to the region involved in SA binding [6], [15]. Recently the crystal structure of the sialic acid – σ1 complex was elucidated [26]. There is a remarkable heterogeneity in the amino acid sequence of the SA-binding domains of different T3D and T1L strains. Some isolates cannot bind SA as determined on JAM-A negative murine erythroleukemia (MEL) cells. The forced selection of such strains yielded mutants that could infect MEL cells probably via the interaction with SA [38].

**Figure 8:**
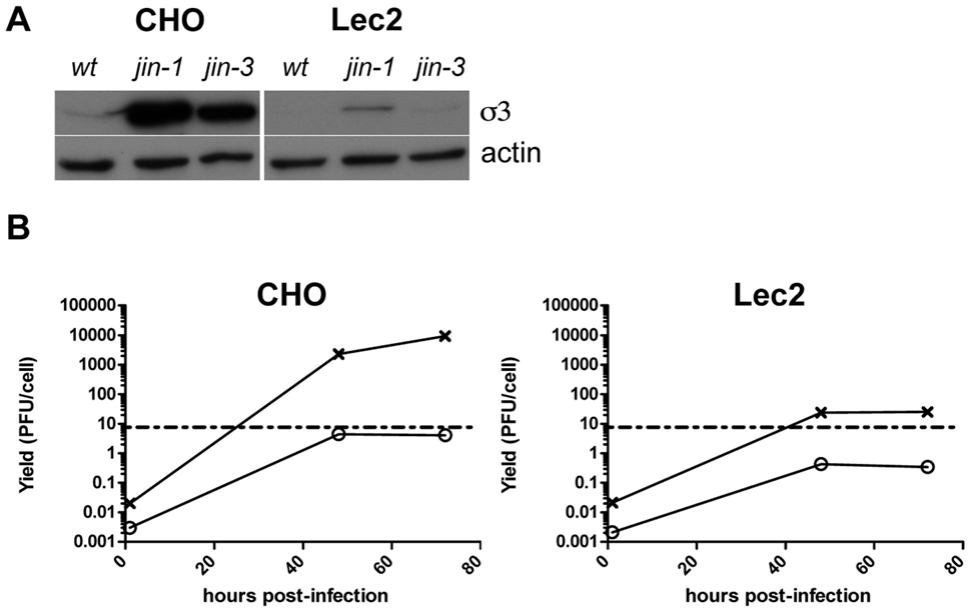
Lec2 cells are poorly infected by reovirus mutants *jin-1* and *jin-3*. (A) CMP-sialic acid transporter defective Lec2 cells and parental cell line, CHO, were exposed to *wt* T3D, *jin-1,* and *jin-3* at MOI of 10. Protein lysates were made 32 hrs post-infection and analyzed by SDS-polyacrylamide gel electrophoresis. For the immunodetection of σ3 the anti-reovirus σ3 antserum 4F2 was used, and anti-actin (human) was used to detect actin as a loading control. (B) Virus production of *wt* T3D and *jin-1* in CHO and Lec2 cells. Cells were exposed for one hour to the viruses at MOI of 10, washed with PBS and immediately lyzed (1 hour time point), or incubated at 37°C for 48 hrs and 72 hrs. Virus yields were determined by plaque assays on 911 cells. The graph shows a representative example of the assay. Open circles: *wt* T3D(o), crosses: *jin-1*(x). The dashed line represents input amount of virus used at the initial infection (10 pfu/cell).

**Figure 9:**
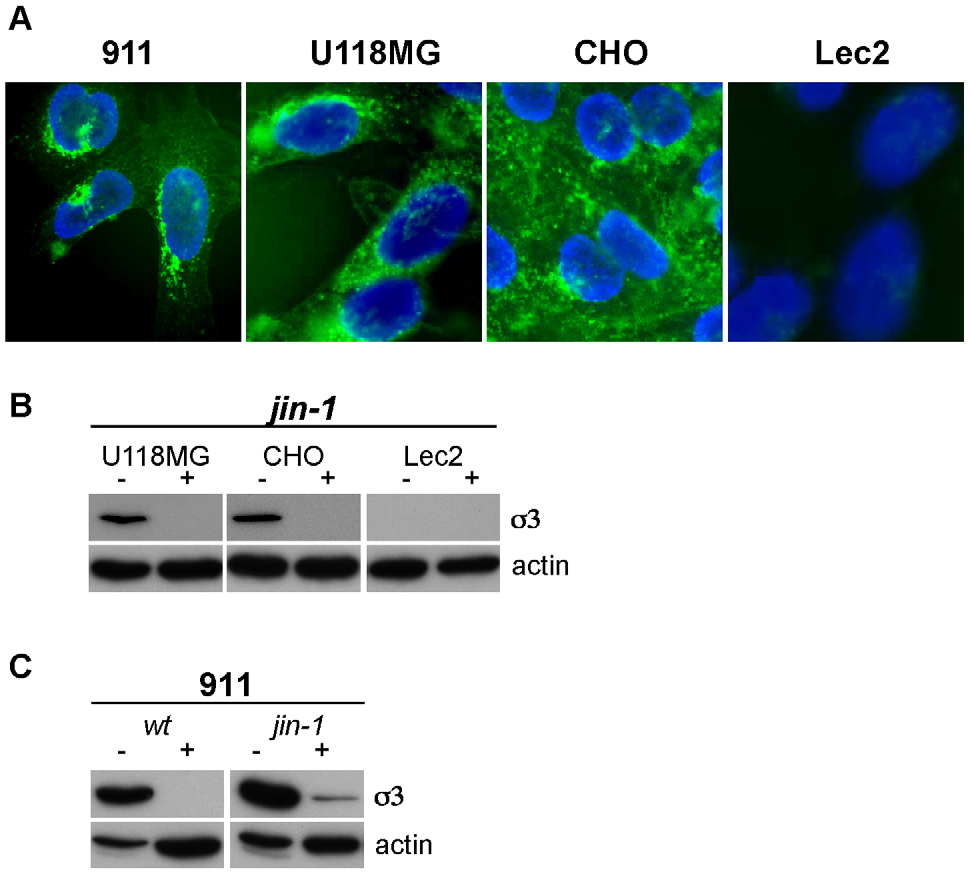
WGA inhibits binding of reovirus to cells. (A) Detection of Sialic acids in cell lines (911, CHO, U118MG and Lec2) by FITC-labeled WGA immunofluorescence. (B) WGA inhibition of reovirus infection. Prior to exposure of reovirus *jin-1*, the cells (U118MG, CHO, and Lec2) were mock-treated (−) or treated with 100 µg/ml WGA for 1 hr. at 37°C (+). After exposure of the cells to the virus at 4°C the cells were washed with PBS and incubated for an additional 32 hours in a CO_2_ incubator before protein lysates were made. For the immunodetection of the σ3 protein the anti-reovirus σ3 (4F2) was used and anti-actin was used as a loading control. (C) WGA inhibition of *wt* T3D and *jin-1* reovirus infection in 911 cells.

The *wt* T3D that was used in our studies has an amino-acid sequence of the sialic-acid binding pocket that is identical to strains capable of binding sialic acid. The S1 mutations found in the *jin* mutants are not located in the region coding for the SA-binding pocket of σ1 (viz. amino acids 198–204; ref [15], [26]), but are located in close proximity of this region. Nevertheless, it is conceivable that the amino acid alterations in the *jin* mutants affect the affinity or avidity of SA binding. To investigate the involvement of SA in binding of our mutant viruses we used Lec2 cells. Lec2 cells have a strongly reduced (by about 90%) amount of sialic acids on their cell surface [39], [40]. Lec2 cells are mutants derived from Chinese Hamster ovary (CHO) cells, which are poorly infected by *wt* T3D reovirus [41], [42]. In contrast, both the *jin-1* and *jin-3* mutants efficiently infect CHO cells (Fig. 8A). The *wt* T3D nor *jin-1* and *jin-3* infect Lec2 cells, as is evidenced by lack of detectable σ3 in the cells exposed to these viruses (Fig. 8A). Also the replication of *jin-1* in the Lec2 cells is markedly reduced compared with the yields obtained in the parental CHO cells (Fig. 8B). This suggests that the expanded tropism of *jin* is dependent on the presence of SA on the cell surface. To support the utilization of SA by the *jin-1* mutant, we shielded the SA on the surface of the cells by pre-incubating the cells with wheat germ agglutinin (WGA). WGA is a lectin with a strong affinity to a broad range of sialoconjugates. To confirm that WGA effectively binds to the cell lines 911, U118MG and CHO, but not to Lec2, we employed FITC-labeled WGA on fixed cells grown on cover slips (Fig. 9A). For the competition experiments we blocked the sialic acids with WGA prior to the binding of *jin-1* to the cells. The addition of WGA to the cells inhibited entry of *jin-1* in U118MG and CHO cells (Fig. 9B). Also in 911 cells, *jin-1* and *wt* T3D infection are inhibited (Fig. 9C). This confirms the dependency of *wt* T3D on SA for cell binding and entry. Our data demonstrate that also the *jin-1* mutant relies on SA binding for cellular entry.

## Discussion

The use of tumor-selective oncolytic viruses for killing tumor cells that resist conventional therapeutic approaches is conceptually attractive. Human reoviruses are one of the promising candidates for use as replicating oncolytic agent [7], [43], [44]. Reoviruses preferentially induce cell death and apoptosis in tumor cells, but not in diploid, non-transformed cells [1]–[3]. However, in some tumor cells expression of the JAM-A receptors is down-regulated and absent on the cell surface, thereby limiting the susceptibility of the cells to reovirus T3D infection.

Here we report the isolation of JAM-A independent T3D reoviruses with an expanded tropism. These mutants, designated as *jin* mutants, may be considered as oncolytic agents in those tumor types that lack accessible JAM-A on their surface [21]–[23], [45]. Although we encountered the first *jin* mutant in a batch of S1-His modified reovirus after selection in the U118MG-scFvHis cell line [24], the *jin* mutants are not genetically modified viruses in the formal sense since they resulted from spontaneous mutations in T3D viruses.

In three independent virus batches we identified *jin* mutants. Two of these (*jin-1* and *jin-2*) carried an identical mutation in the head domain of the spike protein σ1. The mutation replaces the glutamine (Q) residue at position 336 by an arginine (R). The amino acid 336 is located at a surface exposed position, close to the region involved in the trimerization of the σ1 spikes [32]. The alteration renders the area more positively charged. This could potentially result in conformational changes that may disturb the formation of σ1-trimers. However, the results of a trimerization assay (depicted in Fig. 3) revealed that altered σ1 of *jin-1* still forms mature trimers, showing that the Q336R alteration in S1 of the *jin-1* and *jin-2* viruses does not affect trimer formation of this domain.

While the *jin* mutants were isolated on human glioblastoma cell line U118MG, we found that the *jin-1* mutant efficiently infects a variety of reovirus T3D resistant cell lines, including the chicken hepatoma cell line LMH, but not non-transformed primary human skin fibroblasts (VH10) This expanded tropism, together with the small-plaque phenotype observed in the JAM-A positive cell line 911, is reminiscent of changes observed in other virus families. Adaptation to cell culture conditions can result in selection of viruses that acquired the capacity to bind heparan sulfates [46]–[48]. Variants of foot-and-mouth disease virus (FMDV) which bind strongly to heparan sulfate *in vitro*, show small plaques on BHK cells and these variants are attenuated in cattle. Furthermore, they have a decreased ability to spread from the site of inoculation [48]. Alternatively, mutants of Sindbis Virus that exhibit a reduced binding to heparan sulfate give rise to larger plaques *in vitro* and are more virulent *in vivo* with slower clearance from the circulation [46]. However, the Q336R change in *jin-1* and *jin-2* does not yield a typical linear heparin-binding domain consensus -X-B-B-X-B-X- and -X-B-B-B-X-X-B-X- in which B is a basic residue (mainly K or R ) and X a hydropathic residue [49]–[51]. It should be noted that the presence of a linear consensus sequence is not a strict prerequisite for glycosaminoglycan binding. Some of Venezuelan equine encephalitis virus (VEE) mutants bind heparan sulfates (HS) through a conformational domain and do not contain the linear HS-binding domain [52]. Our observation that reovirus *jin-1* entry into U118MG cells cannot be inhibited by incubation with heparin or heparan sulphate (data not shown) suggests that binding to glycosaminoglycans is not responsible for the broadened tropism of *jin-1* and *jin-2.* It therefore remains to be established if and how the Q336R change contributes to the expanded tropism. Also with respect to sialic acid binding, in some DNA viruses mutations in the SA-binding pocket resulted in changes in plaque morphology [53], [54].

Upon continued serial passaging of *jin* mutants in U118MG cells, additional amino acid alterations accumulated in spike protein σ1, T193M in *jin-1* and G187R in *jin-2*. For the *jin-3* mutant, a single mutation in S1 resulted in G196R substitution in σ1. Those changes are located in close proximity of the region implicated in sialic acid binding [6], [15], [25], [26]. This is in line with the previous observation that passaging of reoviruses incapable of binding SA on mouse erythroleukemia cells yielded mutants capable of binding sialic acids [38]. In co-crystalization experiments of σ1 in complex with SA it was shown that the changes were mapped in the σ1 region between amino acids 198 and 204 [26].

Also outer capsid protein σ3 plays an important role in the process of reoviral entry [16], [18], [55], [56]. A mutation found in so-called persistent-infection reoviruses leads to an amino acid change Y354H in the σ3 protein. This alteration has been linked to the reoviral resistance to the protease inhibitor E64d [57]. A mutation in *jin-1* results in M357T in σ3, which is in close proximity of position 354. However, no such mutations are found in *jin -2* and *jin-3*. Moreover, *jin-1* is sensitive to E64d, demonstrating that the *jin-1* virus still depends on cysteine proteases for uncoating and infection. This suggests that the *jin-1* virus enters JAM-A negative cells via the endocytic pathway, like *wt* T3D in JAM-A expressing cells.

So far, we have evaluated the *jin* mutants in *in vitro* studies only and it remains to be elucidated what the effect will be *in vivo*, both with regards to safety, as well as to their oncolytic efficacy.

While we cannot exclude the possibility of the recruitment of secondary receptors, our data suggest that the *jin* mutants rely on sialic acid binding for internalization. It is tempting to speculate that a changed affinity for sialic acids underlies the changed tropism of our *jin* viruses, since they show a decreased ability to spread in cultured cells, exhibit a small plaque phenotype, and shielding SA moieties with WGA prevents the *jin* viruses to enter cells. It remains to be established if the mutation in S1 affects reovirus pathology in mice. In this respect, it is noteworthy that the pathology of reoviruses is, in part, dependent on the σ1 protein [58].

## Materials and Methods

### Cell Lines

Cell lines 911 (generated previously in our lab, see reference [55]), U118MG (obtained from ATCC), U2OS (obtained from ATCC, see reference [29]), CHO (obtained from ATCC), Eoma (obtained from ATCC, see reference [28]) and VH10 (primary human foreskin fibroblasts, provided by B. Klein [59]) were cultured in Dulbeco’s Modified Eagle Medium (DMEM) containing high glucose (Invitrogen, Breda, The Netherlands), supplemented with penicillin, streptomycin (pen-strep) and 8% fetal bovine serum (FBS) (Invitrogen, Breda, The Netherlands). The U118-HAJAM cells were cultured in DMEM plus 8% FBS and pen-strep, supplemented with 200 µg/ml G418. LMH cells (obtained from ATCC, see reference [27]) were grown on collagen I coated dishes (Rat Tail collagen, 2.5 µg/cm^2^, Invitrogen, Breda, The Netherlands) in DMEM plus 8%FBS and pen-strep. STA-ET2.1 cells were grown on collagen I coated dishes (5.0 µg/cm^2^) in RPMI 1640 medium (Invitrogen, Breda, The Netherlands), supplemented with pen-strep and 10% FBS. Lec2 cells (derived from ATCC, see reference [33]) were cultured in alpha-MEM (Invitrogen, Breda, The Netherlands) with 8% FBS. All cells are cultured in an atmosphere of 5% CO_2_ at 37°C.

### Reovirus Propagation

The wild-type T3D virus strain R124 (see accession numbers) was isolated from a reovirus T3D stock obtained from the American Type Culture Collection (stock VR-824) by two rounds of plaque purification on 911 cells. The 911 cells were used to propagate R124 (referred to as *wt* T3D in the text) as described previously [60]. Briefly, cells were exposed to reovirus in DMEM plus 2% FBS for 2 hours at 37°C, 5% CO_2_. Subsequently, the inoculum was replaced by DMEM containing 8% FBS. The virus was harvested 48 hours post infection by resuspending the cells in phosphate-buffered saline (PBS) with 2% FBS and subjecting the suspension to three cycles of freezing and thawing. The sample was cleared by centrifugation for 10 minutes at 800 g. For the mutant *jin* viruses, U118MG cells were used to propagate the viruses after one round of plaque purification on 911 cells. The *jin* viruses were routinely harvested from U118MG cells 72 hours post infection. The experiments were done with virus-containing freeze-thaw lysates, unless otherwise indicated. The infectious reovirus titers of the strains were determined by plaque assay on 911 cells.

### Origin of the *jin*-mutants

*jin-1* is derived from U118scFvHis cells during our experiments on genetically modifying reovirus [24]. The *jin-1* virus was first grown on U118scFvHis cells for two propagations, before three passages on U118MG cells (first point of S1-sequence analysis). The virus was subjected to one round of plaque purification on 911 cells to obtain a homogenous population. This was further propagated on U118MG cells for 11 rounds before analysis of the complete genome sequence (Table 1).

*jin-2* was isolated from U118scFvHis cells infected with *wt* T3D reovirus and passaged twice in these cells. The S1 segment was sequenced from this passage. Subsequently it was passaged 10 times on U118MG cells. After plaque purification, the complete genome sequence was determined (Table 1).

*jin-3* was isolated from U118MG cells exposed to *wt* T3D virus followed by blindly passaging the virus for 6 rounds. From the resulted preparation a virus was isolated by plaque purification and following by 10 additional passages on the U118MG cells. (Table 1).

### Yield Determinations

To determine the replication of *wt*T3D, *jin-1*, *jin-2* and *jin-3* in U118MG and VH10 cells (Fig. 3), cells were seeded in 24-well plate with a cell density of 1*10^5^ cells/well. Viruses (in DMEM plus 2% FBS) were added to the cells with an MOI of 10, two wells per virus. After an exposure of one hour in incubator (37°C, 5% CO_2_) the inoculum was removed and the cells were washed once with PBS and fed by fresh DMEM plus 2% FBS. Reoviruses were harvested from medium and cells by 3 cycles of freeze-thawing, 72 hours after infection. Yields were determined by plaque assays on 911 cells.

For the replication of *wt*T3D or *jin-1* in 911, U118MG, LMH, U2OS, CHO and Lec2 cells (Fig. 7B and 8B), cells were seeded in 6 well plates with a density of 1.5*10^6^ cells/well. Wild-type T3D or *jin-1* was added to 4 wells in case of 911 cells and 3 wells for the other cell lines with an MOI of 10 (in DMEM plus 2%FBS). After one hour of exposure in incubator, the viruses were washed from the cells and medium was replaced. From one well, immediately after washing once with PBS, cells in medium were collected and subjected to freeze-thaw cycles (1 hour time point). 32 Hours (911 cells only), 48 and 72 hours after infection cells and medium were collected and subjected to freeze-thaw cycles. Viral yields were determined by plaque assays on 911 cells.

### Cell Viability Assay

WST-1 reagent (Roche, Almere, The Netherlands) was used to assay the viability of cells after reovirus infections. U118MG and 911 cells in 96-well plate were mock-infected or infected with *wt* T3D or *jin-1* with an MOI of 10, in triplo. Six days post infection WST-1 reagent was added, according to the manufacturer’s manual. The viability measurements in mock-infected cell cultures, were set to 100%.

### [^35^S] Methionine Labelling

Infected cells (911 cells infected at MOI = 1; U118MG and U118-HAJAM with MOI = 5) or mock-infected cells were incubated with TRAN^35^S - LABEL™ (10mCi/ml; MP Biomedicals, Eindhoven, The Netherlands) for 4 h; one day (911 cells) or two days (U118MG and U118HA-JAM cells) post infection. Cells were washed once with phosphate-buffered saline and lysed in Giordano Lysis Buffer (50 mM Tris HCl pH 7.4, 250 mM NaCl, 0.1% Triton, 5 mM EDTA) containing protease inhibitors (Complete mini tablets, Roche Diagnostics, Almere, The Netherlands). The labelling assays were performed in 24-well plates with 5 µl (50 µCi) TRAN35S - LABEL™ per well. The cells were lysed with 100 µl lysis buffer. After addition of sample buffer, 50 microliters per lysate was loaded in the wells of a 10% SDS-polyacrylamide gel. Gels were dried and exposed to a radiographic film to visualize the labeled proteins.

### Immunofluorescence Assay

For immunofluorescence assays, U118MG and 911 cells were grown on glass coverslips in 24-well plates before infection with *wt* T3D or *jin-1* with an MOI of 5 or no virus. One day post infection the cells were fixed with cold methanol (15 minutes, 4°C), washed with PBS containing 0.05% Tween-20, and incubated with antibody 4F2 directed against reovirus σ3 (monoclonal antibody developed by T.S. Dermody [61]; obtained from the Developmental Studies Hybridoma Bank developed under the auspices of the NICHD and maintained by The University of Iowa, Department of Biology, Iowa City, IA 52242), diluted in PBS containing 3% BSA. After incubation at room temperature the cells were washed (PBS, 0.05% Tween-20) and incubated with secondary fluorescein isothiocyanate (FITC)-conjugated goat anti-mouse serum for 30 minutes at room temperature. The mounting solution consisted of glycerol containing 0.02 M Tris HCl pH8.0, 2.3% 1,4-diazabicyclo-[2.2.2]-octane and 0,5 µg/ml 4′,6-diamidino-2-phenylindole (DAPI) to visualize the nuclei.

### RT-PCR and Sequencing

Total RNA was isolated (Absolutely RNA miniprep kit; Stratagene, Agilent Technologies, Amstelveen, The Netherlands) from U118MG cells infected with the different reovirus mutants (*jin-1* or *jin-3*), one day post infection. For *wt* T3D total RNA was isolated from infected 911 cells. Primers used for the RT-PCR procedures are listed in Table S1. DNA synthesis of all the segments started with the unique endR primer designed for every segment, using SuperScript III (Invitrogen, Woerden, The Netherlands) for the reverse transcription process. For the PCR, Pfu polymerase (Promega, Leiden, The Netherlands) was used with the primer combinations unique for every segment. PCR products were first cleaned with Sureclean (Bioline, London, UK), according to the manual, before direct analysis of the sequence. In some cases the resulting PCR products were cloned into plasmid pJet1 (GeneJet, PCR cloning kit; Stratagene, Agilent Technologies, Amstelveen, The Netherlands) and their DNA sequences were determined. All sequence data were generated by The Leiden Genome Center (LGTC, Leiden, The Netherlands).

### *In vitro* Transcription-Translation and Trimerization Assay

All primer sequences can be found in table S1. With DualSHFor and DualSHRev primers, S1 PCR product was generated from plasmid pCDNART3S1 [24], according to manual of the pDual-GC vector (Stratagene, Agilent Technologies, Amstelveen, The Netherlands). The resulting construct (pDualS1His) contained no stop codon, meaning that the Myc-His tag was present behind S1. To introduce a stop codon behind the S1 ORF in pDualS1His, the DualS1st for and rev primers were used in a mutation PCR, with EXL polymerase (Stratagene, Agilent Technologies, Amstelveen, The Netherlands). This resulted in the plasmid pDGC-S1delTag, which was used for the trimerization assay and as start to generate the pDGC-S1QR and pDGC-S1Y313A (with S1-QRmut2Rev and For combi or S1-Y313AmRev and For combi, respectively) also with EXL polymerase. For the *in vitro* transcription-translation (ITT) part, TNT® T7 Quick Coupled Transcription/Translation kit (Promega, Leiden, The Netherlands) was used. Input for the ITT assays were the plasmids pDGC-S1delTag, pDGC-S1QR and pDGC-S1Y313A. The total reaction volume was 15 µl, scaled according to the manual (in the presence of 6 µCi TRAN35S - LABEL™; MP Biomedicals, Eindhoven, The Netherlands). For the trimerization Assay, one fifth of every ITT reaction per construct was used and incubated with Sample buffer (final concentrations: 10% glycerol, 2% SDS, 60 mM Tris HCl pH 6.7, 2.5% β-mercaptoethanol and 2.5% bromophenol blue) for 30 minutes at 37°C to stabilize the trimers or boiled for 5 minutes 96°C to disrupt the trimers. After incubation the samples were loaded on a 10% SDS-polyacrylamide gel, which was kept at 4°C during the run. Gels were dried and exposed to a radiographic film to visualize the labeled proteins.

### Plaque Assay and Size Measurements

Plaque assays were performed in a standard assay as previous described for adenoviruses [62] with minor modifications. Briefly, virus stocks were serial diluted in DMEM containing 2% FBS. The dilutions were added to near-confluent 911 cells in six-well plates. Four hours after infection, medium was replaced with agar-medium. Agar-medium consists of (final concentrations) 0.5% agarose (Ultrapure™, Invitrogen, The Netherlands), 1× minimal essential medium (MEM), 2% FBS, 12.5 mM MgCl2, 2 mM GlutaMAX™ (Invitrogen, Almere, The Netherlands) and 1× pen-strep antibiotic mixture (Invitrogen, Almere,The Netherlands). Plaques are counted six days post infection. Plaques sizes were measured four days post infection. For the measurements a CKX41 Olympus microscope was used and the plaque area was measured with the software of Olympus: Olympus DP-soft.

### Western Analysis

Cell lysates were made in Giordano Lysis Buffer supplemented with protease inhibitors (Complete mini tablets, Roche Diagnostics, Almere, The Netherlands). Total amount of protein in the lysates was measured (Bradford, Biorad, Veenendaal, The Netherlands) and the same amount of lysate (30 µg) was loaded into the wells of a 10% SDS-polyacrylamide gel after addition of western sample buffer (final concentrations: 10% glycerol, 2% SDS, 50 mM Tris HCl pH 6.8, 2.5% β-mercaptoethanol and 0.025% bromophenol blue). The proteins were transferred to Immobilon-P (Millipore, Etten-Leur, The Netherlands) and visualized using standard protocols. Antibodies used in this study: 4F2 directed against reovirus σ3; β-Actin antibody: ImmunO anti-Actin clone C4 (MP Biomedicals, Eindhoven, The Netherlands).

### E64d Inhibition

E64d (Sigma Aldrich, Zwijndrecht The Netherlands) was dissolved in DMSO before use. U118MG and 911 cells were seeded in 24 well plates; half of the cells were exposed to 100 µM E64d at 37°C, 5%CO_2_ for one hour. Purified *wt* T3D or *jin-1* virus and ISVPs (approximately 2*10^3^ particles per cell) were added to the cells and left for one hour at 4°C; cells were washed with PBS and transferred back to 37°C, 5%CO_2_ in the absence or presence of E64d, for 36 hours. Lysates were made as described in Western analysis. For the immunodetection the anti-σ3 antibody (4F2) and β-Actin antibody were used.

### Generation of ISVPs

*wt* T3D or *jin-1* virus ISVPs are freshly prepared by treating CsCl purified virions [60] with chymotrypsin. Purified viruses were diluted to a concentration of 10^11^ PFU/ml in Reovirus Storage Buffer (10 mM Tris HCl pH 7.5, 150 mM NaCl, 10 mM MgCl_2_) and treated with 200 µg/ml Chymotrypsin (TLCK treated, Sigma Aldrich, Zwijndrecht The Netherlands; C3142) at 37°C for 1 hour. Reaction was stopped by adding 5 mM phenylmethyl- sulfonyl fluoride (Sigma Aldrich, Zwijndrecht The Netherlands).

### Wheat Germ Agglutinin (WGA) Binding and Competition Experiment

For the detection of sialic acids in the different cell lines, FITC-labeled WGA (Sigma Aldrich, Zwijndrecht The Netherlands) was used. Cells (grown on round glass coverslips in 24-well plate) were fixed with ice-cold Methanol (15 minutes, 4°C) and washed with PBS containing 0.05% Tween-20. WGA-FITC was added to the cells at a concentration of 5 µg/ml in PBS containing 3% BSA and incubated for 1 hour at room temperature. Excess of unbound WGA-FITC was washed away with PBS containing 0.05% Tween-20. Nuclei were visualized by DAPI in the same mounting solution as is described for the immunofluorescence assay.

WGA competition experiment was done by exposing cells (in 24-well plate wells) to WGA at a concentration of 100 µg/ml in culture medium for one hour in CO_2_ incubator at 37°C. The pre-incubation medium was removed and *wt* T3D or *jin-1* virus was added to the cells with an MOI of 10 in DMEM containing 2% FBS at 4°C for one hour. Cells were washed with ice-cold PBS and normal culture medium was added to the cells. Cells were left for 32 hr in CO_2_ incubator (37°C) before lysates were made as described in Western analysis. For the immunodetection the anti-σ3 antibody (4F2) and β-Actin antibody were used.

### Accession Numbers

GenBank ID’s of wt T3D (R124) and *jin-1* segments:

*R124* T3D-L1 GU991659; *R124* T3D-L2 GU991660; *R124* T3D-L3 GU991661; *R124* T3D-M1 GU991662; *R124* T3D-M2GU991663; *R124* T3D-M3 GU991664; *R124* T3D-S1 GU991665; *R124* T3D-S2 GU991666; *R124* T3D-S3 GU991667; *R124* T3D-S4 GU991668.

*jin-1*-L1 GU991669; *jin-1*-L2 GU991670; *jin-1*-L3 GU991671; *jin-1*-M1 GU991672; *jin-1*-M2GU991673; *jin-1*-M3 GU991674; *jin-1*-S1 GU991675; *jin-1*-S2 GU991676; *jin-1*-S3 GU991677; *jin-1*-S4 GU991678.

## Supporting Information

Table S1 **List of primers used in this study.** First part of the table contains the primers used for the Reverse Transcription PCR experiments. Middle part contains the additional primers used to sequence the different segments. The last part contains the primers used to clone the S1 and S1mutants in the Dual-GC system for the trimerization experiments.
(DOCX)

## References

[1] Coffey MC, Strong JE, Forsyth PA, Lee PW (1998) Reovirus therapy of tumors with activated Ras pathway. Science 282: 1332–1334. 9812900 10.1126/science.282.5392.1332

[2] Norman KL, Lee PW (2005) Not all viruses are bad guys: the case for reovirus in cancer therapy. Drug Discov Today 10: 847–855. 15970267 10.1016/S1359-6446(05)03483-5

[3] Strong JE, Coffey MC, Tang D, Sabinin P, Lee PW (1998) The molecular basis of viral oncolysis: usurpation of the Ras signaling pathway by reovirus. The EMBO journal 17: 3351–3362. 9628872 10.1093/emboj/17.12.3351 PMC1170673

[4] Tyler KL, Fields BN (2001) Mammalian Reoviruses. In: Knipe DM, Howely PM, editors. Philadelphia: Lippincott Williams & Wilkins. 1729–1745.

[5] Barton ES, Chappell JD, Connolly JL, Forrest JC, Dermody TS (2001) Reovirus receptors and apoptosis. Virology 290: 173–180. 11883182 10.1006/viro.2001.1160

[6] Connolly JL, Barton ES, Dermody TS (2001) Reovirus binding to cell surface sialic acid potentiates virus-induced apoptosis. J Virol 75: 4029–4039. 11287552 10.1128/JVI.75.9.4029-4039.2001 PMC114148

[7] Kim M, Chung YH, Johnston RN (2007) Reovirus and tumor oncolysis. JM 45: 187–192. 17618222

[8] Norman KL, Hirasawa K, Yang AD, Shields MA, Lee PW (2004) Reovirus oncolysis: the Ras/RalGEF/p38 pathway dictates host cell permissiveness to reovirus infection. Proc Natl Acad Sci U S A 101: 11099–11104. 15263068 10.1073/pnas.0404310101 PMC503746

[9] Smakman N, Van Den Wollenberg DJM, Borel Rinkes IHM, Hoeben RC, Kranenburg O (2005) Sensitization to apoptosis underlies KrasD12-dependent oncolysis of murine C26 colorectal carcinoma cells by reovirus T3D. J Virol 79: 14981–14985. 16282499 10.1128/JVI.79.23.14981-14985.2005 PMC1287595

[10] Forsyth P, Roldan G, George D, Wallace C, Palmer CA, et al (2008) A Phase I Trial of Intratumoral Administration of Reovirus in Patients With Histologically Confirmed Recurrent Malignant Gliomas. Mol Ther 16: 627–632. 18253152 10.1038/sj.mt.6300403

[11] Vidal L, Pandha HS, Yap TA, White CL, Twigger K, et al (2008) A phase I study of intravenous oncolytic reovirus type 3 Dearing in patients with advanced cancer. Clin Cancer Res 14: 7127–7137. 18981012 10.1158/1078-0432.CCR-08-0524

[12] Gollamudi R, Ghalib MH, Desai KK, Chaudhary I, Wong B, et al (2010) Intravenous administration of Reolysin, a live replication competent RNA virus is safe in patients with advanced solid tumors. Invest New Drugs 28: 641–649. 19572105 10.1007/s10637-009-9279-8 PMC3851036

[13] White CL, Twigger KR, Vidal L, De Bono JS, Coffey M, et al (2008) Characterization of the adaptive and innate immune response to intravenous oncolytic reovirus (Dearing type 3) during a phase I clinical trial. Gen Ther 15: 911–920. 10.1038/gt.2008.21 18323793

[14] O’Donnell SM, Hansberger MW, Dermody TS (2003) Viral and cellular determinants of apoptosis induced by mammalian reovirus. Int Rev Immunol 22: 477–503. 12959755 10.1080/08830180305212

[15] Barton ES, Connolly JL, Forrest JC, Chappell JD, Dermody TS (2001) Utilization of sialic acid as a coreceptor enhances reovirus attachment by multistep adhesion strengthening. J Biol Chem 276: 2200–2211. 11054410 10.1074/jbc.M004680200

[16] Guglielmi KM, Johnson EM, Stehle T, Dermody TS (2006) Attachment and cell entry of mammalian orthoreovirus. Curr Top Microbiol Immunol 309: 1–38. 16909895 10.1007/3-540-30773-7_1

[17] Maginnis MS, Forrest JC, Kopecky-Bromberg SA, Dickeson SK, Santoro SA, et al (2006) Beta1 integrin mediates internalization of mammalian reovirus. J Virol 80: 2760–2770. 16501085 10.1128/JVI.80.6.2760-2770.2006 PMC1395463

[18] Alain T, Kim TS, Lun X, Liacini A, Schiff LA, et al (2007) Proteolytic disassembly is a critical determinant for reovirus oncolysis. Mol Ther 15: 1512–1521. 17519890 10.1038/sj.mt.6300207 PMC7185731

[19] Borsa J, Morash BD, Sargent MD, Copps TP, Lievaart PA, et al (1979) Two modes of entry of reovirus particles into L cells. J Gen Vir 45: 161–170. 10.1099/0022-1317-45-1-161 521802

[20] Mandell KJ, Parkos CA (2005) The JAM family of proteins. Adv Drug Deliv Rev 57: 857–867. 15820556 10.1016/j.addr.2005.01.005

[21] Gutwein P, Schramme A, Voss B, Abdel-Bakky MS, Doberstein K, et al (2009) Downregulation of junctional adhesion molecule-A is involved in the progression of clear cell renal cell carcinoma. Biochem Biophys Res Commun 380: 387–391. 19250634 10.1016/j.bbrc.2009.01.100

[22] Smakman N (2006) Human colorectal liver metastases are resistant to Reovirus T3D and display aberrant localization of the reovirus receptor JAM-1. Towards KRAS-directed therapy, Thesis, University Utrecht.

[23] Naik MU, Naik TU, Suckow AT, Duncan MK, Naik UP (2008) Attenuation of Junctional Adhesion Molecule-A Is a Contributing Factor for Breast Cancer Cell Invasion. Cancer Res 68: 2194–2203. 18381425 10.1158/0008-5472.CAN-07-3057

[24] van Den Wollenberg DJM, Van Den Hengel SK, Dautzenberg IJC, Cramer SJ, Kranenburg O, et al (2008) A strategy for genetic modification of the spike-encoding segment of human reovirus T3D for reovirus targeting. Gen Ther 15: 1567–15678. 10.1038/gt.2008.118 18650851

[25] Chappell JD, Gunn VL, Wetzel JD, Baer GS, Dermody TS (1997) Mutations in type 3 reovirus that determine binding to sialic acid are contained in the fibrous tail domain of viral attachment protein sigma1. J Virol 71: 1834–1841. 9032313 10.1128/jvi.71.3.1834-1841.1997 PMC191253

[26] Reiter DM, Frierson JM, Halvorson EE, Kobayashi T, Dermody TS, et al (2011) Crystal Structure of Reovirus Attachment Protein -σ1 in Complex with Sialylated Oligosaccharides. PLoS Pathogens 7: e1002166. 21829363 10.1371/journal.ppat.1002166 PMC3150272

[27] Morris AP, Tawil A, Berkova Z, Wible L, Smith CW, et al (2006) Junctional Adhesion Molecules (JAMs) are differentially expressed in fibroblasts and co-localize with ZO-1 to adherens-like junctions. Cell Commun Adhes 13: 233–247. 16916751 10.1080/15419060600877978

[28] Barton ES, Forrest JC, Connolly JL, Chappell JD, Liu Y, et al (2001) Junction adhesion molecule is a receptor for reovirus. Cell 104: 441–451. 11239401 10.1016/s0092-8674(01)00231-8

[29] Borsa J, Sargent MD, Lievaart PA, Copps TP (1981) Reovirus: evidence for a second step in the intracellular uncoating and transcriptase activation process. Virology 111: 191–200. 7233831 10.1016/0042-6822(81)90664-4

[30] Van Houdt WJ, Smakman N, van Den Wollenberg DJM, Emmink BL, Veenendaal LM, et al (2008) Transient infection of freshly isolated human colorectal tumor cells by reovirus T3D intermediate subviral particles. Cancer Gene Ther 15: 284–292. 18259212 10.1038/cgt.2008.2

[31] Connolly JL, Dermody TS (2002) Virion disassembly is required for apoptosis induced by reovirus. J Virol 76: 1632–1641. 11799158 10.1128/JVI.76.4.1632-1641.2002 PMC135877

[32] Schelling P, Guglielmi KM, Kirchner E, Paetzold B, Dermody TS, et al (2007) The reovirus sigma1 aspartic acid sandwich: a trimerization motif poised for conformational change. J Biol Chem 282: 11582–11589. 17303562 10.1074/jbc.M610805200

[33] Leone G, Maybaum L, Lee PW (1992) The reovirus cell attachment protein possesses two independently active trimerization domains: basis of dominant negative effects. Cell 71: 479–488. 1423608 10.1016/0092-8674(92)90516-f

[34] Kawaguchi T, Nomura K, Hirayama Y, Kitagawa T (1987) Establishment and characterization of a chicken hepatocellular carcinoma cell line, LMH. Cancer Res 47: 4460–4464. 3607775

[35] Obeso J, Weber J, Auerbach R (1990) A hemangioendothelioma-derived cell line: its use as a model for the study of endothelial cell biology. Lab Invest 63: 259–269. 2166185

[36] Pontén J, Saksela E (1967) Two established in vitro cell lines from human mesenchymal tumours. Int J Cancer 2: 434–447. 6081590 10.1002/ijc.2910020505

[37] Kovar H, Jug G, Aryee DN, Zoubek A, Ambros P, et al (1997) Among genes involved in the RB dependent cell cycle regulatory cascade, the p16 tumor suppressor gene is frequently lost in the Ewing family of tumors. Oncogene 15: 2225–2232. 9393981 10.1038/sj.onc.1201397

[38] Rubin DH, Wetzel JD, Williams WV, Cohen JA, Dworkin C, et al (1992) Binding of type 3 reovirus by a domain of the sigma 1 protein important for hemagglutination leads to infection of murine erythroleukemia cells. J Clin Invest 90: 2536–2542. 1281838 10.1172/JCI116147 PMC443412

[39] Arnberg N, Edlund K, Kidd AH, Wadell G (2000) Adenovirus Type 37 Uses Sialic Acid as a Cellular Receptor. J Virol 74: 42–48. 10590089 PMC111511

[40] Eckhardt M, Gotza B, Gerardy-Schahn R (1998) Mutants of the CMP-sialic acid transporter causing the Lec2 phenotype. J Biol Chem 273: 20189–20195. 9685366 10.1074/jbc.273.32.20189

[41] Danthi P, Hansberger MW, Campbell JA, Forrest JC, Dermody TS (2006) JAM-A-independent, antibody-mediated uptake of reovirus into cells leads to apoptosis. J Virol 80: 1261–1270. 16415003 10.1128/JVI.80.3.1261-1270.2006 PMC1346953

[42] Campbell JA, Schelling P, Wetzel JD, Johnson EM, Forrest JC, et al (2005) Junctional adhesion molecule a serves as a receptor for prototype and field-isolate strains of mammalian reovirus. J Virol 79: 7967–7978. 15956543 10.1128/JVI.79.13.7967-7978.2005 PMC1143703

[43] Kelly K, Nawrocki S, Mita A, Coffey M, Giles FJ, et al (2009) Reovirus-based therapy for cancer. EOBT 9: 817–830. 10.1517/14712590903002039 19527106

[44] Norman KL, Lee PW (2000) Reovirus as a novel oncolytic agent. J Clin Invest 105: 1035–1038. 10772645 10.1172/JCI9871 PMC300841

[45] Koshiba H, Hosokawa K, Kubo A, Tokumitsu N, Watanabe A, et al (2009) Junctional adhesion molecule A [corrected] expression in human endometrial carcinoma. Int J Gynecol Cancer 19: 208–213. 19395995 10.1111/IGC.0b013e31819bc6e9

[46] Byrnes AP, Griffin DE (2000) Large-Plaque Mutants of Sindbis Virus Show Reduced Binding to Heparan Sulfate, Heightened Viremia, and Slower Clearance from the Circulation. J Virol 74: 644–651. 10623725 10.1128/jvi.74.2.644-651.2000 PMC111583

[47] Hulst MM, van Gennip HG, Moormann RJ (2000) Passage of classical swine fever virus in cultured swine kidney cells selects virus variants that bind to heparan sulfate due to a single amino acid change in envelope protein E(rns). J Virol 74: 9553–9561. 11000226 10.1128/jvi.74.20.9553-9561.2000 PMC112386

[48] Sa-carvalho D, Reider E, Baxt B, Rodarte R, Tanuri A, et al (1997) Tissue culture adaptation of foot-and-mouth disease virus selects viruses that bind to heparin and are attenuated in cattle. J Virol 71: 5115–5123. 9188578 10.1128/jvi.71.7.5115-5123.1997 PMC191746

[49] Cardin AD, Weintraub HJ (1989) Molecular modeling of protein-glycosaminoglycan interactions. Arteriosclerosis 9: 21–32. 2463827 10.1161/01.atv.9.1.21

[50] Hileman RE, Fromm JR, Weiler JM, Linhardt RJ (1998) Glycosaminoglycan-protein interactions: definition of consensus sites in glycosaminoglycan binding proteins. BioEssays 20: 156–167. 9631661 10.1002/(SICI)1521-1878(199802)20:2<156::AID-BIES8>3.0.CO;2-R

[51] Vives RR, Lortat-Jacob H, Fender P (2006) Heparan sulphate proteoglycans and viral vectors : ally or foe? Curr Gene Ther 6: 35–44. 16475944 10.2174/156652306775515565

[52] Bernard K, Klimstra WB, Johnston RE (2000) Mutations in the E2 glycoprotein of Venezuelan equine encephalitis virus confer heparan sulfate interaction, low morbidity, and rapid clearance from blood of mice. Virology 276: 93–103. 11021998 10.1006/viro.2000.0546

[53] Bauer PH, Cui C, Liu WR, Stehle T, Harrison SC, et al (1999) Discrimination between sialic acid-containing receptors and pseudoreceptors regulates polyomavirus spread in the mouse. J Virol 73: 5826–5832. 10364334 10.1128/jvi.73.7.5826-5832.1999 PMC112643

[54] López-Bueno A, Rubio MP, Bryant N, McKenna R, Agbandje-McKenna M, et al (2006) Host-selected amino acid changes at the sialic acid binding pocket of the parvovirus capsid modulate cell binding affinity and determine virulence. J Virol 80: 1563–1573. 16415031 10.1128/JVI.80.3.1563-1573.2006 PMC1346950

[55] Chandran K, Nibert ML (2003) Animal cell invasion by a large nonenveloped virus: reovirus delivers the goods. Trends Microbiol 11: 374–382. 12915095 10.1016/s0966-842x(03)00178-1

[56] Jané-Valbuena J, Breun LA, Schiff LA, Nibert ML (2002) Sites and determinants of early cleavages in the proteolytic processing pathway of reovirus surface protein sigma3. J Virol 76: 5184–5197. 11967333 10.1128/JVI.76.10.5184-5197.2002 PMC136125

[57] Wilson GJ, Nason EL, Hardy CS, Ebert DH, Wetzel JD, et al (2002) A single mutation in the carboxy terminus of reovirus outer-capsid protein sigma 3 confers enhanced kinetics of sigma 3 proteolysis, resistance to inhibitors of viral disassembly, and alterations in sigma 3 structure. J Virol 76: 9832–9843. 12208961 10.1128/JVI.76.19.9832-9843.2002 PMC136532

[58] Tyler KL (1998) Pathogenesis of reovirus infections of the central nervous system. Curr Top Microbiol Immunol 233: 93–124. 9599934 10.1007/978-3-642-72095-6_6

[59] Klein B, Pastink A, Odijk H, Westerveld A, van Der Eb AJ (1990) Transformation and immortalization of diploid xeroderma pigmentosum fibroblasts. Exp Cell Res 191: 256–262. 2175267 10.1016/0014-4827(90)90012-y

[60] Smakman N, Van Den Wollenberg DJM, Elias SG, Sasazuki T, Shirasawa S, et al (2006) KRAS(D13) Promotes apoptosis of human colorectal tumor cells by ReovirusT3D and oxaliplatin but not by tumor necrosis factor-related apoptosis-inducing ligand. Cancer Res 66: 5403–5408. 16707468 10.1158/0008-5472.CAN-05-4108

[61] Virgin HW, Mann MA, Fields BN, Tyler KL (1991) Monoclonal antibodies to reovirus reveal structure/function relationships between capsid proteins and genetics of susceptibility to antibody action. J Virol 65: 6772–6781. 1719233 10.1128/jvi.65.12.6772-6781.1991 PMC250764

[62] Fallaux FJ, Kranenburg O, Cramer SJ, Houweling A, van Ormondt H, et al (1996) Characterization of 911: a new helper cell line for the titration and propagation of early region 1-deleted adenoviral vectors. Hum Gen Ther 7: 215–222. 10.1089/hum.1996.7.2-215 8788172

