# Supplementary material for: Isolation of Reovirus T3D Mutants Capable of Infecting Human Tumor Cells Independent of Junction Adhesion Molecule-A
Source: PLoS One. 2012 Oct 24;7(10):e48064. doi: 10.1371/journal.pone.0048064 (PMC3480499; doi:10.1371/journal.pone.0048064)
Supplement: Table S1 — List of primers used in this study. First part of the table contains the primers used for the Reverse Transcription PCR experiments. Middle part contains the additional primers used to sequence the different segments. The last part contains the primers used to clone the S1 and S1mutants in the Dual-GC system for the trimerization experiments. (DOCX) [file pone.0048064.s001.docx]

**Supplementary Data**

***Table S 1. Primers used in this study***

| Name | Sequence (5'-3') | Length |
| --- | --- | --- |
| RT-PCR primers | | |
| S1for | GCTATTGGTCGGATGGATCCTCG | 23 |
| S1end R | GATGAAATGCCCCAGTGC | 18 |
| S2for | GCTATTCGCTGGTCAGTTATGGC | 23 |
| S2endR | GATGAATGTGTGGTCAGTCGTGAGG | 25 |
| S3for | GCTAAAGTCACGCCTGTCGTC | 21 |
| S3endR | GATGATTAGGCGTCACCCACCACCAAG | 27 |
| S4for | GCTATTTTTGCCTCTTCCCAG | 21 |
| S4endR | GATGAATGAAGCCTGTCCCACGTCA | 25 |
| M1for | GCTATTCGCGGTCATGGCTTA | 21 |
| M1endR | GATGAAGCGCGTACGTAGTCTTAG | 24 |
| M2for | GCTAATCTGCTGACCGTTACTC | 22 |
| M2endR | GATGATTTGCCTGCATCCCTTAAC | 24 |
| M3for | GCTAAAGTGACCGTGGTCATGGCT | 24 |
| M3endR | GATGAATGGGGGTCGGGAAGGCTTA | 25 |
| L1for | GCTACACGTTCCACGACAATG | 21 |
| L1endR | GATGAGTTGACGCACCACGACCCATG | 26 |
| L2for | GCTAAATGGCGCGATGGCGA | 20 |
| L2endR | GATGAATTAGGCGCGCTCACGAGGGA | 26 |
| L3for | GCTAATCGTCAGGATGAAGCGG | 22 |
| L3endR | GATGAATCGGCCCAACTAGCATTG | 24 |
| Additional sequence primers | | |
| L1midSQ1 For | GCTAGCTCAAGTTATTCATGGTTTA | 25 |
| L1midSQ2 Rev | GCTATGTCATATTTCCATCCGAATTC | 26 |
| L1midSQ3 For | GTGAAACTATTCAGAACGATCTAG | 24 |
| L1seq3 For | GTCTGGACGAGCGGCCCCT | 19 |
| L1seq4 Rev | CGCGCTTTCTTATCATTGG | 19 |
| L1seq5 For | GTCTGGTAGTGCGGTCATTGAG | 22 |
| L2midFor | GAACAGAAGATCTTGCCCAA | 20 |
| L2midRev | GTCTCGATACCAGTCACGGA | 20 |
| L2seq2 Rev | TGGTATAGATTCCTGCGTCG | 20 |
| L2seq3 For | TGGACGCATGACTCTTCAGC | 20 |
| L2seq4Rev | CTGAGGAAGTTCCGATGAAAGCA | 23 |
| L2sq6For | CTCTAGTGGGATCTAATGCT | 20 |
| L3mdSQ6_REV | CGTTCTGCCATTGTACTGCTG | 21 |
| L3mdSQ7_FOR | GGACTTCACCAATGAGTTAAC | 21 |
| L3midSQ1 For | GTTCAAGTTTCGGCTGATGTCG | 22 |
| L3midSQ2 Rev | CGTGGCCACGTGTGAGGCGTTG | 22 |
| L3seq4 For | ATGACCCTAGCCAACATG | 18 |
| L3seq5 Rev | TGCAGATACATTGGTGTC | 18 |
| L3seq8Rev | CAAATGTGTCGACTGAACACG | 21 |
| M1midSQ1_For | AATCTTGTTATATGCTCC | 18 |
| M1midSQ2_Rev | TGAGCGAATGTTAGCAATC | 19 |
| M1midSQ3_Rev | CATGATTTGGATTCCTAAT | 19 |
| M2midFor | GGAACTAATTGGCATCTCAA | 20 |
| M2midRev | TTGCCGTTTGGATCCCAGCT | 20 |
| M3midSQ1 For | GATCCTGAAATCTATAACGAG | 21 |
| M3midSQ2 Rev | CTGCTGCAACACAGGACATTC | 21 |
| M3midSQ3_For | TTGCTTGACGCTGTGCGTGTCG | 22 |
| S4midFor | AGCCTTAAACCTGATGATCG | 20 |
| S4midRev | AGAATTGGGTTTGACGAGCA | 20 |
| Dual-GC primers | | |
| DualS1st rev | AGCGCGGCCGCAGTGTTTAAACTTCACGTGAAACTACGCGGGT | 43 |
| DualS1st for | ACCCGCGTAGTTTCACGTGAAGTTTAAACACTGCGGCCGCGCT | 43 |
| DualSH rev | GCCTCTTCTAAGCGTGAAACTACGCGG | 27 |
| DualSH for | GCCTCTTCCATGGATCCTCGCCTACGT | 27 |
| S1-QRmut2Rev | AATGTCGGAGTTCACCCGTACCCTCCAATTCAGC | 34 |
| S1-QRmut2For | GCTGAATTGGAGGGTACGGGTGAACTCCGACATT | 34 |
| Y313A S1mrev | GCTCTGCCTAAACCTAGCATTTGGACTCATTCCGATA | 37 |
| Y313A S1mfor | TATCGGAATGAGTCCAAATGCTAGGTTTAGGCAGAGC | 37 |
